# Pre-dauer starvation decouples somatic from germline development with lifelong reproductive consequences in *C. elegans*

**DOI:** 10.1101/2025.05.23.655783

**Published:** 2025-05-28

**Authors:** Fred A. Koitz, Camille P. Miller, Kacy Lynn Gordon

**Affiliations:** 1 Department of Biology, The University of North Carolina at Chapel Hill, Chapel Hill, North Carolina 27599

**Keywords:** *C. elegans*, dauer, starvation, LAG-2, germline, germline stem cell niche

## Abstract

Early life stresses impact reproductive outcomes in many organisms. In response to crowding and starvation, *C. elegans* nematodes form dauer larvae, in which development arrests until conditions improve. We discovered dramatic differences in gonad size and germ cell number among dauers that form under different conditions. We used live cell imaging of fluorescent markers in otherwise wild-type and mutant animals combined with food-removal, recovery, and brood-size assays to investigate the causes and consequences of this germline difference. Pre-dauer feeding, but not nutrient sensing via the DAF-2/insulin-like signaling receptor or DAF-7/TGF-β, is required for plasticity in gonad size. Gonad differences in dauer have lifelong reproductive consequences; worms with small dauer gonads recover to have smaller broods. Somatic and germline development are decoupled as pre-dauer starvation induces germline quiescence while the soma continues its development until dauer is formed. A rapid return to germline Notch-dependence and an increase in presentation by the germline stem cell niche of the Notch ligand LAG-2–regulated at the protein and not transcript level–are among the earliest events of dauer recovery.

## SUMMARY STATEMENT

Pre-dauer starvation limits the size of the dauer gonad, reduces presentation of the germline niche cue LAG-2, and diminishes future reproductive success.

## INTRODUCTION

Lifelong development requires environmental responsiveness within the developmental program. A special case of developmental response to difficult environmental conditions is the diapause state, in which development arrests in unfavorable conditions and resumes when conditions improve (Harsimran Kaur Gill et al., 2017; Renfree and Fenelon, 2017). While diapause states can be obligate - that is, built into an animal’s life cycle in anticipation of poor conditions - other diapause states are facultative and responsive to an organism’s present environment (Hand et al., 2016; Wilsterman et al., 2021). One well-studied facultative diapause state is the *C. elegans* dauer (Cassada and Russell, 1975), formed in response to starvation and crowding sensed by pheromone (Golden and Riddle, 1984a) and enhanced by higher temperatures (Ailion and Thomas, 2000). Dauer larvae withstand poor environmental conditions (Cassada and Russell, 1975; Golden and Riddle, 1984a) for many times the typical lifespan of a worm (Klass and Hirsh, 1976). Improved environmental conditions trigger exit from dauer, resumption of development, and a (mostly) normal reproductive life (Cassada and Russell, 1975; Klass and Hirsh, 1976). Famously, time spent in the dauer larval stage does not affect adult lifespan (Klass and Hirsh, 1976).

It is the preservation of fertility rather than increased lifespan alone that makes dauer adaptive (Schluter et al., 1991). Previous studies disagree about the long-term effects of dauer for future reproduction (Kim and Paik, 2008; Klass and Hirsh, 1976; Ow et al., 2018; Webster et al., 2018). These studies focused on recovery and did not investigate differences of the cells of the dauer reproductive system itself. We set out to investigate the cells of the gonad and germline during the dauer larval period to look for developmental antecedents of later reproductive differences.

To introduce our study, we present brief summaries of the parallel processes of the dauer decision and early reproductive system development. The *C. elegans* life cycle goes from embryonic development through four larval stages (L1-L4) punctuated by molting before a terminal molt into the adult reproductive form (Corsi et al., 2015). The dauer is an alternate third larval stage. Entry into the dauer developmental pathway is specified in the first larval stage (L1) and is followed by a second stage called L2d (Golden and Riddle, 1984a). In persistently harsh conditions, the L2d molts into a dauer larva with a thick cuticle, arrested development, and altered metabolism (Cassada and Russell, 1975; Erkut and Kurzchalia, 2015; Golden and Riddle, 1984a; Wadsworth and Riddle, 1989).

The *C. elegans* hermaphrodite reproductive system develops post-embryonically from a 4-cell primordium of two somatic blast cells and two primordial germ cells (Kimble and Hirsh, 1979). In the L1 stage, a germline stem cell niche, the distal tip cell (DTC) is born from each of the somatic blast cells and the primordial germ cells make a first division or two in the L1 larval stage (Kimble and Hirsh, 1979). The germline relies on DTC expression of the stemness cue LAG-2, a Delta/Serrate/LAG-2 (DSL) ligand of the Notch signaling pathway to maintain germ cells in an undifferentiated state throughout development (Kimble and Crittenden, 2005). By the end of the L2 stage a population of 20–30 germ cells (Kimble and White, 1981) is supported by two DTCs and a 10-cell somatic gonad primordium (Kimble and Hirsh, 1979). The developmental arrest of gonad and germ cell lineages in the dauer larva have been investigated genetically (Narbonne and Roy, 2006; Tenen and Greenwald, 2019), primarily using genetic mutants that constitutively form dauers at high temperature.

Among these are mutations to genes in the insulin-IGF-1 signaling (IIS) pathway, including the gene encoding the sole *C. elegans* IIS receptor *daf-2* (Kimura et al., 1997). IIS integrates nutritional status with metabolism, developmental control, reproduction, and aging in *C. elegans* (Murphy and Hu, 2013) and in other organisms, including humans (Barbieri et al., 2003; Claeys et al., 2002; Das and Arur, 2017). A reduction-of-function allele, *daf-2(e1370),* is commonly used to induce dauer larvae at high temperature in experimental settings (Karp, 2018). Another reduction-of-function allele, *daf-7(e1372)*, affecting a parallel pathway leading to dauer entry–TGF-β signaling–also forms dauers constitutively at high temperature (Karp, 2018). The TGF-β pathway links external cues to the reproductive system (Dalfó et al., 2012; Pekar et al., 2017). The germline is especially sensitive to starvation throughout life (Angelo and Van Gilst, 2009; Dalfó et al., 2012; Pekar et al., 2017; Seidel and Kimble, 2011; Webster et al., 2022), so we hypothesized that pre-dauer nutrition may cause differences in the dauer reproductive system that impact post-dauer recovery of fertility.

We discovered that *C. elegans* enter dauer with different numbers of germline cells depending on feeding prior to dauer entry. The most severely starved worms have many times fewer cells and roughly half the brood size upon recovery compared to those that fed the most during pre-dauer development. Surprisingly, the DAF-2/IIS receptor is dispensable for this difference, suggesting that the fed state itself, rather than nutrient sensing via IIS, regulates pre-dauer germline growth. Because the somatic lineage is invariant in *C. elegans* (Kimble and Hirsh, 1979; Sulston and Horvitz, 1977; Sulston et al., 1983), early life starvation decouples germline from somatic development, pausing germline development while the soma continues to the “safe harbor” state of dauer diapause. The mechanism of this decoupling involves reduced presentation of the stemness cue LAG-2 by the germline stem cell niche and the induction of a Notch-independent quiescent state of the germline. This state is rapidly reversed in the DTC and germline upon removal from dauer-forming conditions, and appears to be regulated post-translationally. Altogether, this work reports heretofore unrecognized differences in dauer gonads mediated by pre-dauer nutrient stress effects on niche and stem cell maintenance that predict differential reproductive recovery from dauer.

## RESULTS

### Dauers that form constitutively have longer gonads than those that form after progressive crowding and starvation

We examined the gonad in dauer animals under two dauer-induction regimes that are commonly used in the field–facultative dauer entry after starvation and crowding and constitutive dauer forming mutants (Karp, 2018). First, we progressively starved crowded worm growth plates (see Methods) and assayed both “early dauers” on such a plate within the first week of starvation, as well as “late dauers” that were recovered after >4 weeks. We used otherwise wild-type worms expressing markers of the germline (*mex-5p::H2B::mCherry::nos-2 3’UTR*, (Linden et al., 2017)) and an endogenously tagged integrin alpha subunit *ina-1(qy23[ina-1::mNG])*(Jayadev et al., 2019)) that aids visualization of the somatic gonad.

We also used the field-standard dauer-formation constitutive (daf-c) mutants carrying reduction-of-function alleles of *daf-2(e1370)* and *daf-7(e1372)*, which enter dauer constitutively at high temperature (Karp, 2018). It is thought that DAF-2/IGFR (insulin-like growth factor receptor) or DAF-7/TGF-β function is impaired, respectively, at all rearing temperatures, as non-dauer-related loss-of-function phenotypes are observed at both low and high temperatures (Blackwell et al., 2015; Gems et al., 1998; Greer et al., 2008; Kenyon et al., 1993; McGehee, 2019; Trent et al., 1983; Tullet et al., 2008). Indeed, dauer itself is triggered by high-temperature rearing in wild-type worms (Ailion and Thomas, 2000), so these daf-c mutants are considered to be reduction-of-function alleles that are sensitized for dauer formation at a higher temperature (Golden and Riddle, 1984b).

This experimental regimen allowed us to compare between early and late dauers, among genotypes after progressive starvation (control with gonad fluorescent markers compared to *daf-2(e1370)* and *daf-7(e1372)*), and between modes of dauer induction for both mutant genotypes (progressive starvation vs. constitutive entry, for both early and late dauers).

**Figure 1:**
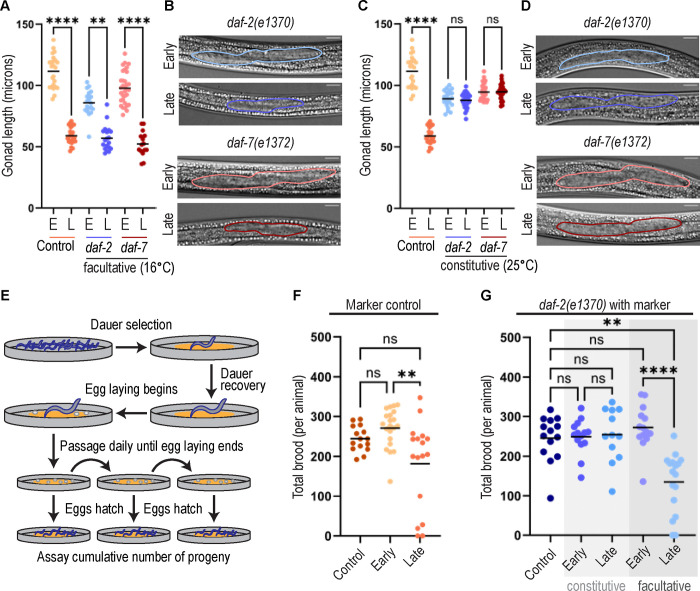
Dauer larvae that form after prolonged starvation and crowding have smaller gonads and recover to have smaller broods than dauer larvae that form constitutively. (A) Gonad length in facultative dauers of control *ina-1::mNG; naSi2(mex-5p::H2B::mCherry::nos-2 3’UTR)* early (n=24) and late (n=28), *daf-2(e1370);naSi2(mex-5p::H2B::mCherry::nos-2 3’UTR)* early (n=19) and late (n=19), *and daf-7(e1372)* early (n=33) and late (n=15). Kruskal-Wallis test statistic=109.2, p<0.0001 with Dunn’s correction for multiple comparisons. (B) Representative differential interference contrast (DIC) microscopy images of facultative mutant early and late dauer gonads. Gonads outlined. (C) Gonad length in worms reared at 25°C. Control dataset same as in (1A), *daf-2(e1370);naSi2(mex-5p::H2B::mCherry::nos-2 3’UTR)* constitutive early (n=22) and late (n=29) dauers, and *daf-7(e1372)* constitutive early (n=31) and late (n=32) dauers. Kruskal-Wallis test statistic=109.1, p<0.0001 with Dunn’s correction for multiple comparisons. (D) Representative DIC images of constitutive mutant early and late dauer gonads. Gonads outlined. (E) Schematic for brood size counts in (1F,G). (F) Brood size of control strain. Never-dauer control (n=15), recovered early (n=21), and recovered late (n=17). Includes animals that died after recovery and animals that were fully sterile, does not include animals that failed to recover from dauer. Kruskal-Wallis test statistic=10.89, p=0.0043 with Dunn’s correction for multiple comparisons. (G) Brood size of *daf-2(e1370);naSi2(mex-5p::H2B::mCherry::nos-2 3’UTR)*. Never-dauer control (n=14), light shaded background indicates constitutive early (n=14) and late (n=12) recovered animals, dark shaded background indicates facultative early (n=14) and late (n=17) recovered animals. Includes animals that died after recovery and animals that were fully sterile but not animals that failed to recover from dauer. Kruskal-Wallis test statistic=27.76, p<0.0001 with Dunn’s correction for multiple comparisons. All scale bars 10 μM. Significance indicated as p<0.0001 ****; p<0.005 **.

Under progressive starvation, early dauers of all three genotypes had longer gonads than late dauers, by nearly 2x on average (Fig. 1A,B). This raised the possibility that dauer gonads change over time. We tested this hypothesis by using the dauer constitutive mutants to form a population of constitutive, synchronous dauers and measuring both early and late dauers for gonad size. Neither mutant showed a time-related change of gonad length over a month in dauer (Fig. 1C,D), suggesting that time spent in dauer was not responsible for the smaller gonads seen in control late dauers. We therefore concluded that neither genotype (*daf-2(e1370)* or *daf-7(e1372)* vs. control) nor time in dauer (over one month) is responsible for the gonad size difference between early and late dauers formed by progressive starvation.

### Worms with longer gonads have better post-dauer reproductive recovery than worms with shorter gonads, regardless of genotype

We next asked if these same dauer-forming conditions for the same genotypes considered above had functional consequences for post-dauer reproductive success. We recovered dauers (Methods) and tracked individual worm recovery and eventual brood size (Fig. 1E). For each genotype, we included a never-dauer, continuously-fed control, since brood size can vary across strains. Otherwise wild-type worms with fluorescent markers (see next section) show a normal brood size (of ~244 ± 31) in never-dauer controls. Early dauers induced by progressive starvation (which have long gonads, Fig. 1A) recover to produce a normal brood (271 ± 47) (Fig. 1F Early). In contrast, late dauers (which have small gonads, Fig. 1A) recover to produce a substantially decreased brood size compared to the early dauers (171 ± 112) (Fig. 1F Late). The same pattern is observed for the *daf-2(e1370)* mutant genotype after progressive starvation: early dauers (long gonads, Fig. 1A,B) produce normal broods relative to the never-dauer control, while late dauers (short gonads, Fig. 1C,D) have worse reproductive recovery (Fig. 1G).

When mutant *daf-2(e1370)* animals with the germ cell nuclear marker constitutively entered dauer (larger germlines, Fig. 1C,D) both early and late dauers recovered equally well, both producing broods equivalent to the never-dauer control of that genotype (Fig. 1G). Therefore, time spent in dauer alone did not reduce the brood of subsequently recovered dauer animals. From these brood size assays, we concluded that dauer gonad size was best correlated with post-dauer reproductive success. We next asked what determines dauer gonad size.

Two of our prior experiments and one published finding (Cassada and Russell, 1975) implicate pre-dauer feeding as a potential determinant of gonad size in dauer. First, when we used daf-c alleles to induce constitutive dauer formation at high temperature, the worms feed continuously until right before dauer entry and had uniformly large gonads and brood. Second, for all genotypes exposed to progressive starvation, early dauers from newly starved plates had large gonads and broods, and may have had access to more food during pre-dauer development than late dauers. Finally, dauer body length differs proportionally with the titre of bacterial food that worms ate prior to dauer (Cassada and Russell, 1975). We hypothesized that pre-dauer feeding determines gonad size in dauer.

### Starvation in the second larval stage decouples germline development from somatic development independent of insulin-like signaling

**Figure 2:**
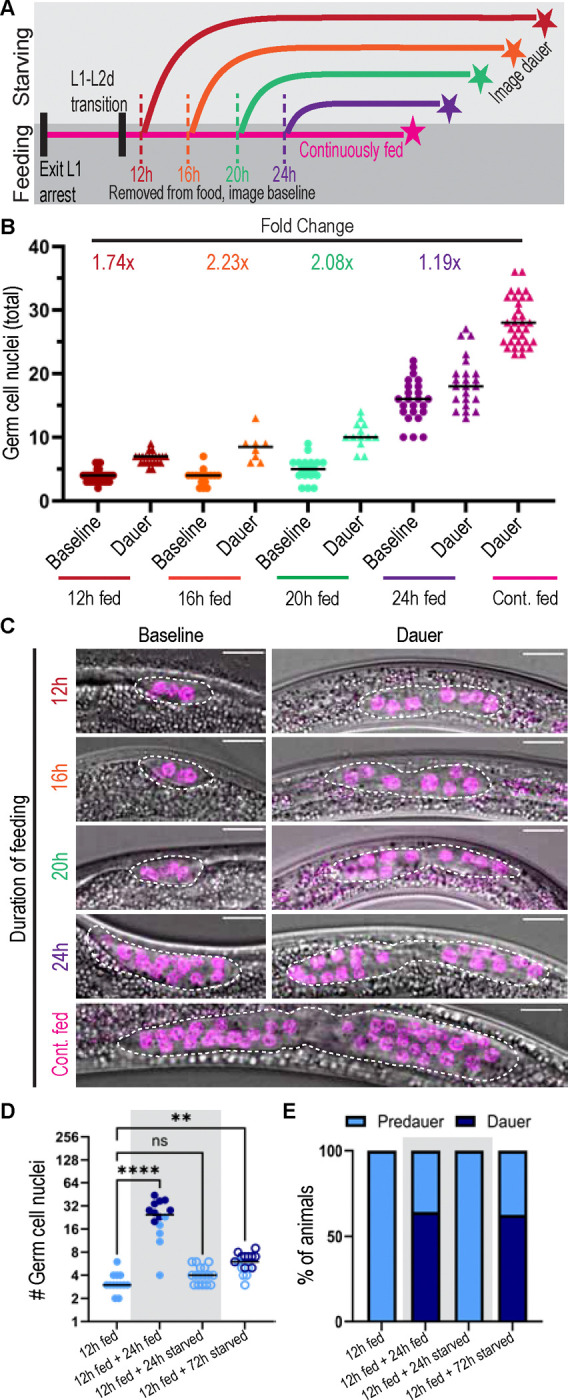
Withdrawal of food in the pre-dauer period arrests germ cell proliferation after a single round of division. (A) Schematic illustrating food removal experiments. Worms moved off food (dashed lines, imaged “Baseline” (2B,C)) and reared until dauer entry (stars, imaged “Dauer” (2B,C). All experiments in Fig. 2 were conducted at 25°C. (B) Results of experiment detailed in (2A), showing number of germ cell nuclei in *daf-2(e1370);naSi2(mex-5p::H2B::mCherry::nos-2 3’UTR)*. Sample sizes: 12h fed baseline n=31, dauers n=22; 16h fed baseline n=16, dauers n=8; 20h fed baseline n=18, dauers n=13; 24h baseline n=23, dauer=24; continuously fed dauers n=33. (C) Representative images of *daf-2(e1370);naSi2(mex-5p::H2B::mCherry::nos-2 3’UTR)* (magenta) measured for (2B) at time of food removal (baseline, left) and in dauer (right). Gross structure of the gonad outlined in white. (D) Experiment as in (2A) with additional +24h endpoint. Darker dots indicate animals in dauer diapause at the time of counting (see 2E). “12h fed” n=13; “12h fed, 24h starved” n=16; “12h fed, 24h fed” n=14, refers to animals that were washed at 12h and returned to food. “12h fed, 72h starved” n=16. Kruskal-Wallis test statistic=39.12; p<0.0001 with Dunn’s correction for multiple comparisons. (E) Percentage of worms that had reached the dauer stage under each condition in 2D. In 2D,E, a grey shaded box marks the samples quantified in parallel after 24 hours under experimental conditions, both on and off food. All scale bars 10 μM. Significance indicated as p<0.0001 ****; p<0.005 **.

To test the hypothesis that feeding is responsible for dauer gonad length, we manipulated *daf-2(e1370)* daf-c mutants expressing a germ cell nuclear marker (*mex-5p::H2B::mCherry::nos-2 3’UTR*, (Linden et al., 2017)) in a series of food removal experiments. The use of a daf-c mutant at 25°C ensured that we could vary food exposure while ensuring every animal was in the L2d dauer entry program. Worms were reared on growth plates with a lawn of bacterial food for the specified number of hours at 25°C (Fig. 2A), removed from food at 25°C, and maintained until the endpoint at which a population of majority dauers was noted.

We assayed germ cell number directly upon removal from food and again within a day of dauer entry to test the relationship between time spent feeding and germ cell number, and how much germ cell number changed between the cessation of feeding and entry into dauer (Fig. 2B). We found a proportional relationship between time spent feeding and germ cell number at the time of food removal and in dauer.

Between removal from food at the 12, 16, and 20 hours fed timepoints, it appears that cells undergo one additional round of division before dauer entry (average germ cell numbers approximately double, Fig. 2B,C). After 24 hours or longer of L2d feeding, the increase in cell number before dauer entry is less dramatic, likely reflecting that late L2d animals begin to slow germ cell division in preparation for dauer entry ((Narbonne and Roy, 2006), also in *daf-2(e1370)* mutants). Germ cells will complete an average of one additional cell division after DTC laser ablation in the L2-L3 stages of development (Kimble and White, 1981), and genetic ablation of the stemness receptor GLP-1/Notch allows germ cells to complete their current mitotic cell cycle and make a terminal division before differentiating (Fox and Schedl, 2015). Our finding that germ cells complete one round of division after food removal (Fig. 2B,D) tracks with our established understanding of cell cycle constraints on stem-like germ cell proliferative dynamics.

Comparing the rate of germline growth among these treatments revealed an even more dramatic response to starvation than that of germ cell number alone (Fig. 2D). Animals that fed (12h fed+24h fed) gained an average of about one germ cell per hour, while animals that starved (12h fed+24h starved) added only one germ cell in that 24 hour period (Fig. 2D, gray shaded box). In response to starvation, animals are able to arrest germline development while slowing but not arresting somatic development until the dauer larva is formed (Fig. 2E and (Cassada and Russell, 1975)), demonstrating a decoupling of germline development from somatic development during the L2d period. Because these experiments were done in *daf-2(e1370)* mutants, another major conclusion from this experiment is that DAF-2/IGFR-mediated nutrient sensing itself is not required for worms to modulate gonad size in response to food removal.

### Dauer gonad size differences are driven by germline cell number and somatic cell size

**Figure 3:**
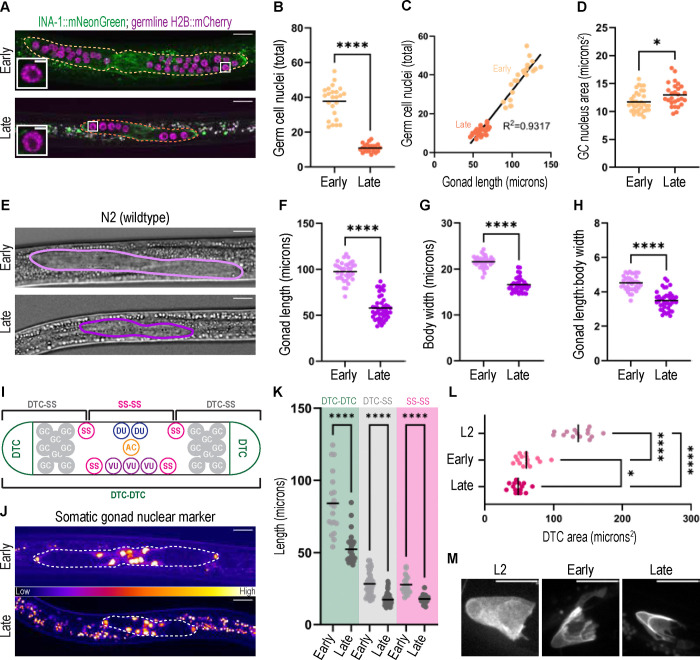
Early and late dauers differ in somatic gonad cell size and germline cell number (A) Representative images of early and late dauers expressing (green) *ina-1::mNG*, an alpha integrin subunit and (magenta) *naSi2(mex-5p::H2B::mCherry::nos-2 3’UTR*) transgene marking germ cell nuclei. Gonads outlined. Inset image of a germ cell nucleus showing characteristic compacted chromatin morphology of a quiescent germ cell (Belew et al., 2021), scale bar 2 μM. (B) Germ cell nuclei per animal in *ina-1::mNG;naSi2(mex-5p::H2B::mCherry::nos-2 3’UTR)* early (n=24) and late (n=28) dauers. Mann-Whitney test statistic U=0, p<0.0001. (C) Germ cell number (3B) is highly correlated to the length of the gonad (same samples as in Fig. 1A) for early (light) and late (dark) dauers. R^2^=0.9317. (D) Nuclear size of early (n=35 nuclei from 3 worms) and late (n=27 nuclei from 4 worms) dauers shown in 3A,B. Unpaired t-test statistic t=2.591, df=60; p=0.0120. (E) Representative DIC images of wild type early and late dauer gonads (outlined). (F) Gonad length of wild type early (n=35) and late (n=40) dauers. Mann Whitney test statistic U=18, p<0.0001. (G) Body width of wild type early (n=35) and late (n=40) dauers (same samples as in 3F). Mann Whitney test statistic U=17, p<0.0001. (H) Test for isometric scaling of gonad with body. Ratio of gonad length to body width of the same early (n=35) and late (n=40) dauers in 3F,G. Mann-Whitney test statistic U=111, p<0.0001. (I) Illustration of the 12 somatic gonad cells based on (Lints and Hall, 2004), L2/L3 molt). We observe the same number of cells in the dauer (K, L). Distal tip cells (DTC), sheath-spermathecal (SS), dorsal uterine (DU), ventral uterine (VU), anchor cell (AC). Germ cells (GC) are separated into two gonad arms. Brackets at top indicate measurements used in (3K). (J) Representative images of early (top) and late (bottom) dauer gonads (outlined) with a somatic gonad-specific nuclear marker *arTi435(rps-27p::2xnls::gfp(flexon)::unc-54 3′ UTR); arTi237(ckb-3p::Cre(opti)::tbb-2 3′ UTR)* (Shaffer and Greenwald, 2022)). Single-slice images colored by fire LUT. (K) Distance between three sets of cells of the somatic gonad in early and late dauers of the strain in (3J). DTC-DTC, DTC-closest SS cell in that same arm, and SS-SS cell across the central region. DTC-DTC, Mann-Whitney test statistic U=31, p<0.0001; DTC-SS, Mann-Whitney test statistic U=193, p<0.0001; SS-SS, Mann-Whitney test statistic U=21, p<0.0001. (L) Area of trace around sum projection of DTC as in (M) in early and late dauers and an L2 control. Welch’s ANOVA test statistic=91.91 (2.000, 24.57), p<0.0001 with Dunnett’s T3 post-hoc multiple comparisons test. (M) Sum projection through ten 0.3 μm z-slices of the superficial surface of a DTC expressing a membrane-localized transgenic reporter *qIs154(lag-2p:: myr::tdTomato)* for stages described at top. All scale bars (except inset) 10 μM. Significance indicated as p<0.0001 ****; p<0.005 **; p<0.05 *

In the otherwise wild-type strain expressing *ina-1::mNG* and the germ cell nuclear marker, early dauers had not only longer gonads, but more germ cells than late dauers (Fig. 3A,B), demonstrating that the trend we observe in *daf-2(e1370)* mutants (Fig. 2B,D) holds. Gonad length is highly correlated to germ cell number (Fig. 3C). Late dauer germ cells had slightly larger nuclei (Fig. 3D), and all dauer germ cell nuclei had a compacted chromatin appearance (Fig. 3A, inset) characteristic of germ cell quiescence that has been observed in starvation-induced L1 arrest (Belew et al., 2021).

We next asked if germ cell number was the only factor contributing to gonad size differences. After progressive starvation, early wild-type (N2) dauers (Fig. 3E) displayed greater gonad length (~1.7x) (Fig. 3F) and body width (~1.3x) (Fig. 3G) than late dauers. However, the ratio of gonad length to body width also differs, with the early dauer gonads being proportionally larger compared to the body width (~4.5:1) than those of late dauer gonads (~3.5:1) (Fig. 3H), violating an assumption of simple isometric scaling between body and gonad size. This suggests that growth is regulated differently in the gonad vs. the rest of the body.

Unlike the germline, in which cell divisions vary, the somatic gonad cell lineage is invariant (Kimble and Hirsh, 1979; Sulston and Horvitz, 1977) (Fig. 3I). When we examine a somatic gonad nuclear marker (Shaffer and Greenwald, 2022), early and late dauers have the same number of somatic gonad cells (two DTCs, four sheath-spermathecal (SS) cells, and six uterine blast cells Fig. 3J). We further quantified the size of the somatic gonad as the distance between these nuclei. Early dauers have longer individual gonad arms (DTC-SS) than late dauers in addition to an overall greater gonad length (DTC-DTC) (Fig. 3K). The SS-SS measurements represent the length of the bulk of the somatic gonad blast cells found between the two gonad arms that will give rise to the somatic gonad sheath, spermatheca, and uterus; early dauers were also larger than late dauers by this measure (Fig. 3K). Therefore, late dauers have a more compact somatic gonad than early dauers.

The only somatic gonad cell to be terminally differentiated at this stage of development is the DTC, which is the germline stem cell niche that maintains germ cells in an undifferentiated, stem-like state (Crittenden et al., 2006; Hubbard, 2007; Kimble and White, 1981; Kimble, 2014). Late dauers had smaller DTCs than early dauers, though both had markedly smaller DTC cell size than a continuously-fed L2 control (Fig. 3L,M). Thus, we find two separate mechanisms responsible for the smaller size of the gonad of late dauers, corresponding to their respective cell lineage limitations: while the germline is reduced in cell number, the somatic gonad appears to be more compact with the same number of cells, and the DTCis smaller in cross-section. Because the DTC is required for germ cell proliferation, we next examined the role of this cell in supporting the dauer germline.

### The dauer germline is maintained in a Notch-independent, quiescent state

One necessary factor regulating germline growth is LAG-2 ligand expressed by the DTC activating GLP-1/Notch signaling in the distal germline (Henderson et al., 1994). Under replete conditions, niche signaling via LAG-2 to the GLP-1/Notch receptor in the germ cells is required for their maintenance in a mitotic, undifferentiated state at all developmental stages; both DTC ablation (Kimble and White, 1981) and genetic ablation of Notch signaling (Fox and Schedl, 2015; Kodoyianni et al., 1992) are sufficient to trigger meiosis within hours. However, starvation has been shown to induce a G2-arrested, Notch-independent quiescent state in transiently starved adults (Seidel and Kimble, 2011). We found that primordial germ cells in L1 arrested animals, which are known to be G2-arrested and quiescent (Belew et al., 2021), are also Notch-independent (n=15/15 recovered *glp-1(bn18)* L1 arrested animals were fertile after 24h at 25°C).

We hypothesized that the germline of both early and late dauers, which are in a *de facto* starved state and known to be G2-arrested (Narbonne and Roy, 2006), is similarly Notch-independent. We investigated this question using worms carrying a temperature sensitive allele of *glp-1*/Notch*, glp-1(bn18),* which at the permissive temperature (16°C) have adequate Notch signaling to remain fertile, but upon shifting to the restrictive temperature (25°C) early in development become sterile due to failure of germline induction or irreversible meiotic entry of germ cells (Fox and Schedl, 2015; Kodoyianni et al., 1992). In adults, signs of meiotic entry appear in distal adult germ cells within 6 hrs (Fox and Schedl, 2015; Kodoyianni et al., 1992).

**Figure 4:**
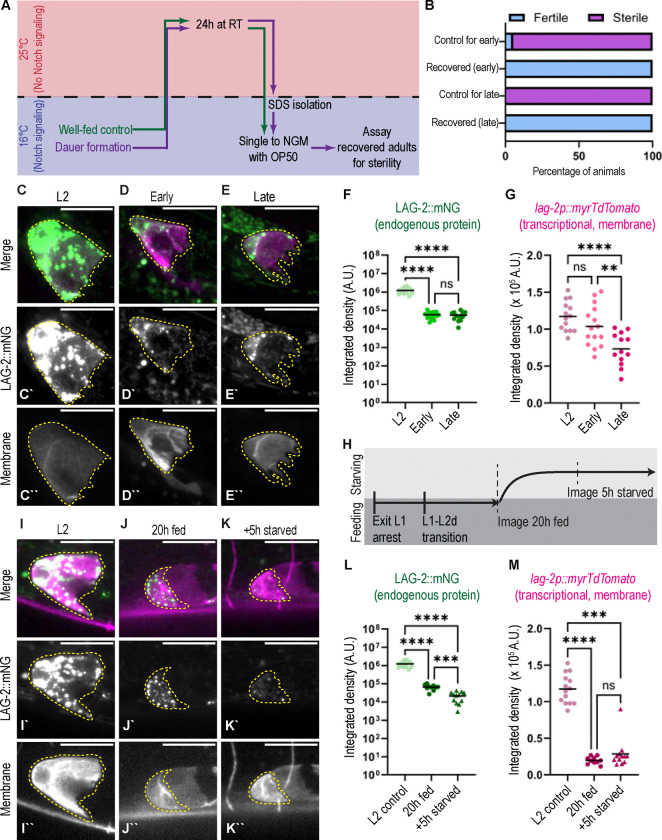
Dauer germ cells are arrested in a Notch independent state with a reduced DTC presentation of the LAG-2 stemness cue (A) Schematic illustrating temperature shift experiment testing germline *glp-1*/Notch-independence in dauer. Well-fed control *glp-1(bn18)* mutants were compared dauers of that same genotype from starved and crowded plates. The experiment was repeated for early (<5d in dauer) and late (>4 weeks) dauers. (B) Worm fertility after (4A). Control (never-dauer population temp shift in parallel with early dauer) n=19, Recovered (early) n=14, Control (temp shift in parallel with late dauer) n=9, Recovered (late) n=15. (C-E) Sum projection through ten 0.3 μm z-slices of the superficial surface of a DTC expressing endogenously tagged LAG-2 protein (green, *lag-2(cp193[lag-2:: mNeonGreen])*) and a membrane-localized transgenic reporter (magenta, *qIs154(lag-2p:: myr::tdTomato)*) for stages described at top. Each fluorescence channel is shown with identical scaling between treatments, meaning apparent differences in brightness reflect actual differences in signal intensity. (C-C”) Fed L2 control. (D-D”) Early dauer. (E-E”) Late dauer. (F-G) Integrated density measured for DTCs described in 4C-E. (F) LAG-2::mNG in early (n=15 DTCs) and late (n=13 DTCs) dauers and L2 control (n=14 DTCs). Welch’s ANOVA test statistic W=43.65 (2.000, 22.50), p<0.0001 with Dunnett’s T3 multiple comparisons. (G) *lag-2p::myrTdTomato* transcriptional reporter in early (n=15 DTCs) and late (n=13 DTCs) dauers and L2 control (n=14 DTCs). Ordinary one-way ANOVA test statistic F=12.27, p<0.0001 with Tukey’s multiple comparisons test. (H) Schematic of experiment performed for 4I-K. The transgenes shown in 4C-E were crossed into a *daf-2(e1370)* mutant background. Worms were raised on food at 25°C for 20h, imaged, moved off of food, and imaged 5h later. (I-K) Representative images of worms described in (4H). Sum projection through ten 0.3 μm z-slices of the superficial surface of the DTC coexpressing endogenously tagged LAG-2 protein (green) with DTC membrane-localized transcriptional reporter (magenta). (I-I”) Fed L2 control animal reared at 25°C. (J-J”) L2d fed for 20h at 25°C. (K-K”) Same as in (J) but 5h after food removal at 25°C. (L-M) Integrated density measured for DTCs described in 4H-K. (L) LAG-2::mNG, 20h L2d fed baseline (n=8) 5h starved (n=12), L2 control (same dataset as shown in (4F)). Welch’s ANOVA test statistic W (DFn, DFd)=61.10 (2.000, 17.93), p<0.0001 with Dunnett’s T3 multiple comparisons. (M) Transcriptional reporter *lag-2p:: myr::tdTomato,* 20 h L2d fed baseline (n=8 DTCs) and 5h starved (n=12), L2 control (same dataset as in (4G)). Kruskal-Wallis test statistic=25.41, p<0.0001 with Dunn’s post-hoc correction for multiple comparisons. All scale bars 10 μM. DTCs outlined in yellow. Significance indicated as p<0.0001 ****; p<0.0005 ***; p<0.005 **.

When continuously-fed populations of *glp-1(bn18)* mutants were shifted to the restrictive temperature for 24 hours (Fig. 4A, and see Methods), nearly all animals singled after the treatment were sterile when shifted back to the permissive temperature (Fig. 4B), as expected. In comparison, when *glp-1(bn18)* dauers–both early and late–were shifted to the restrictive temperature for 24 hours, all recovered as fertile adults at the permissive temperature (Fig. 4B). From this, we conclude that both the early and late dauer germline is in a Notch-independent, quiescent state.

### Starvation reduces presentation of the stemness cue by the germline stem cell niche prior to dauer entry

It is not known whether or how LAG-2 protein abundance or localization changes under conditions that induce a Notch-independent, quiescent germline. We used a strain co-expressing an endogenously tagged LAG-2::mNeonGreen (Gordon et al., 2019) and DTC membrane marker (*lag-2p::myrTdTomato,* (Byrd et al., 2014)) to quantify LAG-2 abundance in an L2 control compared to both early and late dauers. Both early and late dauers had severely diminished (~10x) LAG-2::mNG signal in the DTC compared to the L2 control but did not significantly differ from one another (Fig. 4C–F). The transcriptional reporter is less active in both dauers compared to the L2, but not by the same magnitude as the LAG-2::mNG (Fig. 4B and 4G). While dauer DTCs produce less *lag-2* gene product than fed L2s, the absolute amount of LAG-2 signaling protein in early vs. late dauer cannot explain the different germline sizes of these groups.

We next turned to the dynamics with which LAG-2 expression is diminished during the pre-dauer period, the time during which feeding changes germline growth (shown in Fig. 2B,C). We combined the LAG-2::mNG endogenous protein tag and the *lag-2p::myrTdTomato* transcriptional reporter with the *daf-2(e1370)* allele and performed a timed food removal assay on L2d daf-c animals at 25°C (Fig. 4H, as in Fig. 2). We found that LAG-2::mNG protein abundance is highly responsive to the onset of starvation in L2d. LAG-2::mNG drops from the 20h fed baseline within 5 hours of food removal (Fig 4I–L). Transcription of *lag-2* is known to be lower in late larval animals in unfavorable conditions (low food, high pheromone) that cause reduced DAF-7 signaling (Dalfó et al., 2012; Pekar et al., 2017), and we find that animals in L2d have dramatically less transcription from this reporter than control L2s (Fig. 4M). However, the transcriptional reporter stays fairly steady upon food removal in L2d (Fig. 4M). Thus, reduced *lag-2* transcription cannot be the entire mechanism behind the drop in LAG-2::mNG abundance upon food removal in L2d; diminished LAG-2::mNG protein stability (at least in comparison to the myristoylated TdTomato reporter) must also play a role.

We conclude that dauer germline is maintained in a Notch-independent state with low levels of the stemness cue LAG-2 expressed by the DTC. The amount of LAG-2 being presented to the germ cells does not differ between early and late dauers, however, suggesting that niche limitations in dauer do not regulate the different gonad sizes between these two cohorts. Instead, the timing at which LAG-2 drops is acutely driven (within 5 hours) by the termination of pre-dauer feeding. We next examined the dynamics of Notch signaling during dauer exit.

### Post-dauer Notch-dependence is restored in tandem with a transcription-independent burst of LAG-2 ligand presentation by the germline stem cell niche

We hypothesized that the post-dauer germline would rapidly return to a Notch-dependent state upon germ cell-cycle re-entry during dauer recovery, as proliferating *C. elegans* germ cells are Notch-dependent in other life stages (Austin and Kimble, 1987; Cinquin et al., 2010). We predicted that LAG-2 protein abundance would rapidly increase to meet a post-dauer requirement for Notch signaling. We measured expression in an otherwise wild-type strain of the LAG-2::mNG protein and a *lag-2*-promoter-driven transcriptional reporter upon dauer recovery (see Methods).

**Figure 5:**
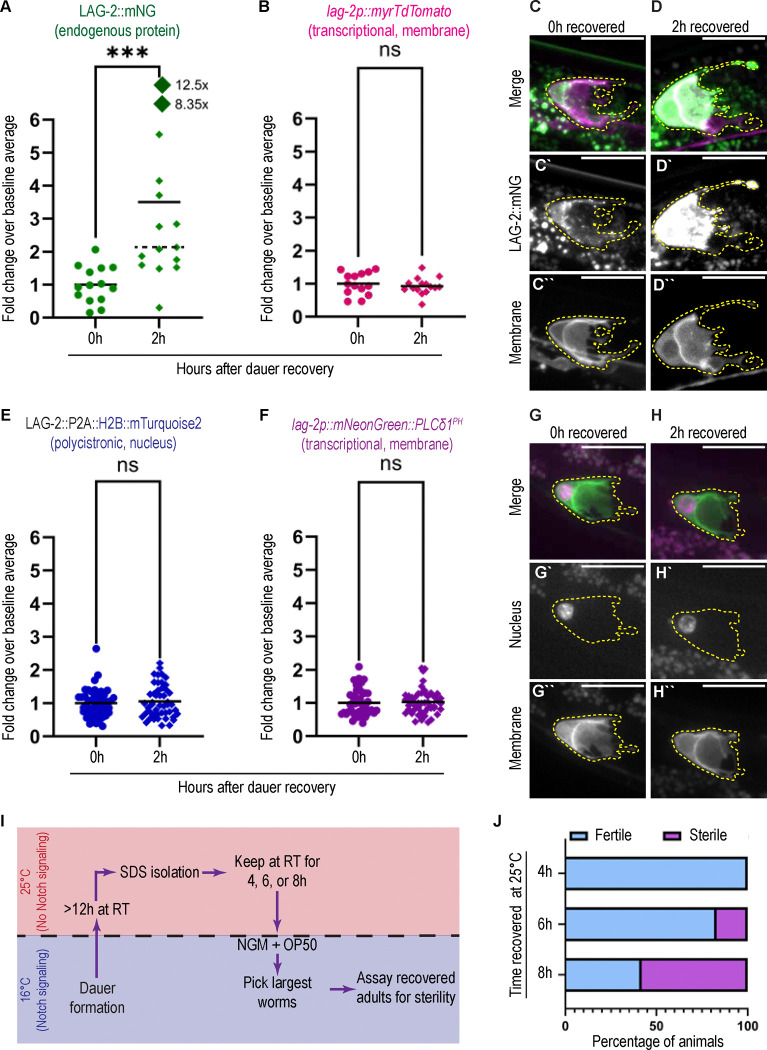
Removal from dauer maintenance conditions triggers a burst of LAG-2 presentation on the DTC niche that precedes a return to Notch dependence (A-D) An otherwise wild-type strain coexpressing the endogenously tagged LAG-2 protein *lag-2(cp193[lag-2:: mNeonGreen])* (green in 5C,D) with a membrane-localized transgenic reporter *qIs154(lag-2p:: myr::tdTomato)* (magenta in 5C,D) in dauer (same dataset at “late” in Fig. 4F,G as this was an extension of that experiment and 2 h after removal from dauer maintenance conditions. (A) Fold change of LAG-2::mNG relative to mean at 0h (0h n=13; 2h n=15). Two high outliers shown at non-proportional distance from the bulk of the data. 2h mean (solid), median (dashed). Mann-Whitney test statistic U=22, p=0.0001. (B) Fold change of the *lag-2p::myrTdTomato* transcriptional reporter for samples in (5A). Unpaired t-test statistic t=0.6645, df=27; p=0.5120 (ns). (C-D) Sum projection through ten 0.3 μm z-slices of the superficial surface of a DTC for stages described at top. For 5C,D, each fluorescence channel is shown with identical scaling between time points, meaning apparent differences in brightness reflect actual differences in signal intensity. (C-C”) 0h of recovery. (D-D”) 2h of recovery. (E-H) Otherwise wild-type strain co-expressing a *lag-2(bmd202* [*lag-2::P2A::H2B::mTurquoise2]*) polycistronic histone reporter knocked into the endogenous *lag-2* locus (magenta in 5G-H) with a *lag-2p::mNeonGreen::PLCδ1*^*PH*^ membrane-localized transcriptional reporter (green in 5G-H), same treatment at A-D. (E) Fold change of polycistronic reporter *lag-2(bmd202* [*lag-2*::P2A::H2B::mTurquoise2]) relative to mean at 0h (0h n=46; 2h n=48). Unpaired t-test statistic t=0.5458, df=92, p=0.5865 (ns). (F) Fold change of *lag-2p::mNeonGreen::PLCδ1*^*PH*^ transcriptional reporter relative to mean at 0h (0h n=46; 2n=48). Mann-Whitney test statistic U=1031, p=0.5851 (ns). (G-H) Sum projections through ~20 0.3 μm Z-slices from superficial surface of DTC to deep bottom of nucleus. Acquired with a 1.6x optical zoom. (G-G”) 0h of recovery. (H-H”) 2h of recovery. (I) Schematic of dauer recovery *glp-1(bn18)* temperature-shift experiment testing the return of Notch-dependence to the germline shown in 5J. (J) Percentage of animals recovering as fertile vs. sterile results after removal from dauer-maintenance conditions at 25°C for the first 4 h (top, n=39), 6 h (middle, n=34)) and 8 h (bottom, n=43) of recovery. DTCs outlined in yellow. All scale bars 10 μM.

At 0 hours of recovery, late dauers had detectable signal of both the LAG-2::mNG protein and the *lag-2* transcriptional reporter (Fig. 5A–D), suggesting that the dauer DTC continues to express the *lag-2* locus and localize LAG-2 protein to the membrane. Dauer expression of *lag-2* reporters in the DTC has been reported previously in *daf-2(e1370)* mutant dauers (Narbonne and Roy, 2006), and here we show that this expression endures through several weeks of dauer. During the first two hours of dauer recovery, LAG-2::mNG protein abundance increases 2–3-fold (Fig. 5A). Surprisingly, we did not observe a corresponding increase in signal from the co-expressed *lag-2p::myrTdTomato* transcriptional reporter (Fig. 5B). Such dynamics suggest that the immediate restoration of LAG-2 on the DTC–much like its initial downregulation upon starvation (Fig. 4I–M)–is post-transcriptionally regulated.

Before drawing this conclusion, we needed to rule out experimental artifacts that could complicate the analysis of the transcriptional reporter. We considered that slow folding of TdTomato fluorescent protein could cause a lag in its transcriptional readout (see Methods), or that post-transcriptional regulation of *lag-2* mRNA could uniquely affect LAG-2 production. We therefore examined the expression of another pair of DTC-expressed markers: a faster-folding, membrane-localized *lag-2p::mNeonGreen::PLCδ1*^*PH*^ (*cpIs122,* (Linden et al., 2017)) and a histone H2B::mTurquoise2 knocked into the endogenous *lag-2* locus with a 2A peptide cleavage site (Medwig-Kinney et al., 2022). This second element will generate a polycistronic mRNA, the translation of which will produce one LAG-2 protein and one H2B::mTurquoise2, meaning that the histone reporter is under the same transcriptional and post-transcriptional, transcript-based regulation (e.g. by 3’ UTR-mediated repression by microRNAs or RNA binding proteins, transcript decay, etc.) as the endogenous *lag-2* gene. In the two hours after recovery from dauer, we observe no change in the levels of expression of either the transcriptional membrane (Fig. 5E,G) or polycistronic nuclear reporter (Fig. 5F,H). We conclude that the *lag-2* locus is transcriptionally active in the DTC during dauer–despite the germline being maintained in a Notch-independent state–and LAG-2 is under protein-specific negative regulation that is rapidly alleviated upon dauer exit.

Given the rapid return of LAG-2 to the DTC surface after dauer exit, we predicted that the germline would become Notch-dependent in the first hours of recovery from dauer. We performed temperature shift experiments during dauer recovery for *glp-1(bn18)* mutant worms (Fig. 5I). Dauer *glp-1(bn18)* mutant animals all recover fertility after an excursion to the restrictive temperature (Fig. 4B). If worms are kept at the restrictive temperature for the first 4 hours of recovery from dauer, the stem-like germ cells remain Notch-independent and the animals recover as fertile adults (Fig. 5J, top, n=39/39). After 6 hours of recovery from dauer at 25°C, ~20% of worms become sterile in adulthood (Fig. 5J, middle, n=7/34). After 8 hours of recovery from dauer at 25°C, more than half of worms will recover as sterile adults (Fig. 5J, bottom, n=25/43). Taken together we see a rebound of LAG-2 protein on the DTC within two hours of recovery from dauer, preceding a return of Notch dependence in the germline between 4–8 hours of recovery. Because dauer exit is asynchronous among individuals (Cassada and Russell, 1975), the precise timing of these events with respect to dauer exit and to one another may be even more tightly coordinated. Other early features of dauer exit–loss of the buccal plug and resumption of pharyngeal pumping–also occur within a two-hour window of removal from dauer conditions (Cassada and Russell, 1975), making the return of LAG-2 on the DTC surface as rapid a response to the end of dauer as any other measured event. This correlation suggests that systemic recovery is more integrated across body systems than pre-dauer changes, which were able to be uncoupled. Our findings establish germline regulation as a central, early feature of dauer recovery.

## DISCUSSION

Here we report that changes in the germline stem cell niche cue LAG-2 are among the earliest responses both to starvation preceding dauer entry and to environmental cues that allow dauer exit. Reduction of pre-dauer feeding reduces the amount of LAG-2 presented by the germline stem cell niche and the proliferation of germline cells. Starved pre-dauer germ cells swiftly become quiescent and complete a single terminal division before dauer entry. The response at the level of germ cell number is dramatic–the most severely starved dauers (wild-type late dauers and *daf-2(e1370)* mutants starved early in L2d) have as few cells in the germline (~5–8) as worms with severe loss-of-function alleles of *glp-1*/Notch receptor gene (Austin and Kimble, 1987), and even fewer germ cells than worms have after early laser ablation of the DTC (Kimble and White, 1981).

While reduced food intake also slowed the rate of somatic progression towards the L2d/dauer molt, it does not alter somatic cell numbers in the somatic gonad (Fig. 3). Instead, the soma as a whole continues its development through L2d and into dauer while the germline arrests, both in terms of absolute germ cell number and–even more dramatically–rate of germline grown over time (Fig. 2).

Differences in germline and somatic response to the starvation-induced L1 larval arrest stage have previously been observed, both in differential transcriptional responses (Webster et al., 2022), and chromatin compaction in the germline (Belew et al., 2021; Morao and Ercan, 2021). The L1 arrest state is triggered by complete starvation after hatching (Baugh, 2013; Johnson et al., 1984). Several checkpoints triggered by later starvation arrest somatic development at a later larval stage transition (Schindler et al., 2014), and the somatic gonad relays environmental information to the germline throughout life (Gordon, 2020). We observe that the soma and germline can decouple their development plastically and rapidly in response to changing environmental conditions in the middle of a larval stage.

Notch signaling via *lag-2* in the nervous system is required both during dauer and for dauer recovery (Ouellet et al., 2008). LAG-2 protein downregulation in the DTC has been observed during worm aging (Aprison et al., 2024 preprint; Singh et al., 2024), and *lag-2* transcription is downregulated via reduced TGF-β signaling after food removal in late larval worms (Dalfó et al., 2012; Pekar et al., 2017). The transition of the germline from a Notch-dependent to a Notch-independent state has been reported during starvation (transient adult starvation (Seidel and Kimble, 2011) and L1 arrest (this paper)), and it should be investigated whether DTC-expressed LAG-2 is also reduced in those contexts. The question remains whether a reduction in LAG-2 signal in response to starvation is the proximal cause of germline quiescence, or whether germline quiescence is a necessary precondition for the germline to tolerate a reduction in LAG-2 as a byproduct of starvation.

Notably, expression of both endogenously tagged LAG-2 protein and *lag-2* promoter-driven transgenes (Fig. 5 and (Narbonne and Roy, 2006)) remain detectable in dauer–even after an an extended duration of dauer diapause–so the downregulation of *lag-2* during starvation is relative and not absolute. The regulation of LAG-2 in the DTC in our experiments also appears to entail post-translational downregulation upon starvation and during dauer which rapidly ceases at dauer exit.

It has previously been shown that the TGF-β pathway regulates *lag-2* expression in the DTC as part of environmental sensing, including as a response to dauer pheromone and low food, though this work was done in late larvae that had bypassed the dauer decision (Pekar et al., 2017). TGF-β signaling is restored during dauer exit (Ren et al., 1996; Schackwitz et al., 1996), so we predict that *lag-2* transcription eventually rises during recovery from dauer, but apparently not on the rapid time scale of the recovery of LAG-2 protein in the DTC.

While the precise molecular mechanisms linking nutrition to LAG-2 protein expression in the DTC remain to be found, we establish that they proceed even with the field-standard *daf-2(e1370)* class 2 loss-of-function allele of the sole *C. elegans* IIS receptor. Not only is starvation-triggered LAG-2 downregulation DAF-2-independent, but the reproductive consequences of pre-dauer food intake are also the same in *daf-2(e1370)* animals as they are in wild-type worms. Worms that fed before dauer (both early dauers of both genotypes and mutant worms induced to form dauer at high temperature) have normal brood sizes after recovery.

The germline is the only lineage in *C. elegans* with an indeterminate division pattern and variable cell number. It is therefore the only cell population that can respond to starvation by limiting the number of cells it produces. We have demonstrated that this unique flexibility allows germline development to be decoupled from the rest of the dauer entry program earlier than was previously thought. In genetic backgrounds in which animals are able to feed before genetically-induced dauer entry, it has been established that the germline and somatic gonad require AMPK (*lbk-1* and *aak-2*) signaling and DAF-18/PTEN–independent of the DAF-2 IIS receptor–to enter a quiescent state (Narbonne and Roy, 2006; Tenen and Greenwald, 2019). Prior to dauer entry, the temperature-sensitive *glp-1* allele in *daf-2(e1370)* genetic background shifted to the restrictive temperature causes germ cell pachytene arrest during dauer (Narbonne and Roy, 2006). Since these *daf-2(e1370)* mutants feed before entering dauer, this shift to the restrictive temperature occurred during what our work suggests is a Notch-dependent phase of pre-dauer germline growth. We find that after starvation prior to dauer entry, the germline undergoes a reduction in the stemness cue presented by the somatic gonad (also independent of DAF-2) before becoming Notch-independent in dauer.

Early life stresses are known to affect later life outcomes in a range of organisms and contexts with potential mechanisms ranging from epigenetics to inflammation to hormone signaling to lasting variation in central nervous system development and function (Taylor, 2010). In *C. elegans,* there is precedent for differential reproductive recovery after dauer depending on whether starvation or crowding (high levels of the dauer pheromone) was the primary trigger to dauer entry (Ow et al., 2018). Our progressive starvation protocol involves both starvation and crowding, and future work will probe the contributions of both factors to pre-dauer germline quiescence.

Taken together, we propose that germline regulation is a key response to both the cues that induce dauer and the withdrawal of those cues in recovery from dauer. Future work will focus on how the germline transitions from a Notch-independent state back to Notch-fueled germline proliferation. One possible driver is nucleotide levels, which have been shown to limit cell division but not cell growth in unicellular organisms (Diehl et al., 2022) and to affect germline growth specifically in non-dauer *C. elegans* (Chi et al., 2016). The rapid restoration of LAG-2 protein to the DTC surface upon withdrawal of dauer-maintenance conditions (Fig. 5) certainly suggests that reestablishing a stem cell niche capable of supporting germline growth is an early and crucial feature of dauer recovery. Our analyses of endogenously tagged LAG-2::mNeonGreen protein recovery vs. transcriptional reporters and a polycistronic reporter altogether suggest that the ligand’s increase at the membrane is regulated post-translationally (Fig. 5). Notch signaling is regulated at the protein level in multiple ways (Fortini, 2009; Suarez Rodriguez et al., 2023; Tian et al., 2004; Weinmaster and Fischer, 2011; Wu et al., 2016; Yamamoto et al., 2010), and future work will delve into this aspect of its regulation upon starvation and during dauer exit. Tellingly, in our experiment, LAG-2 increases upon recovery from dauer in the absence of food, suggesting that LAG-2 is actively inhibited by the dauer pheromone rather than actively promoted by nutritional cues.

Such a mechanism of rapid ligand protein up-regulation by means of alleviated repression could theoretically yield a faster response than upregulation that requires new transcription initiation. However, continuous transcription, translation, and LAG-2 protein degradation comes with an energy cost that a dauer worm would only incur if the alternative–slower restoration of LAG-2 protein on the stem cell niche surface–carried an even greater cost of reduced fertility upon return of the germline to Notch-dependence.

## MATERIALS AND METHODS

Sections of this text are adapted from prior Gordon lab publications (Li et al., 2022; Singh et al., 2024), as they describe our standard laboratory practices and equipment.

### Strains

We used Wormbase (Sternberg et al., 2024) while conducting this study. Some strains were provided by the CGC, which is funded by NIH Office of Research Infrastructure Programs (P40 OD010440) and are to be requested directly from CGC. The following *C. elegans* strains were obtained from the CGC: N2 (Brenner, 1974), CB1370 *daf-2(e1370)* III (Kimura et al., 1997), CB1372 *daf-7(e1372)* III (Vowels and Thomas, 1992), DG2389 *glp-1(bn18)* III (Kodoyianni et al., 1992).

The following *C. elegans* strains were generously shared by community members or generated previously in our lab: NK2517 *qIs154(lag-2p:: myr::tdTomato); lag-2(cp193[lag-2:: mNeonGreenˆ3xFlag])* V) (Gordon et al., 2019), KLG034 *cpIs122(lag-2p::mNeonGreen:: PLCδ1*^*PH*^*)*II*; lag-2(bmd202* [*lag-2*::P2A::H2B::mTurquoise2^lox511Î2xHA]) V. (Singh et al., 2024), GS9692 *arTi435(rps-27p::2xnls::gfp(flexon)::unc-54 3′ UTR* I); *arTi237(ckb-3p::Cre(opti)::tbb-2 3′ UTR* X) (Shaffer and Greenwald, 2022).

The following *C. elegans* strains were generated for this paper by crossing alleles from sources that are cited after each strain description: KLG047 *ina-1(qy23[ina-1::mNeonGreen])*;*naSi2*(*mex-5p::H2B::mCherry::nos-2 3’UTR)* II (Jayadev et al., 2019; Linden et al., 2017), KLG050 *daf-2(e1370)* III*; naSi2(mex-5p::H2B::mCherry::nos-2 3’UTR)* II, KLG051 *daf-2(e1370)* III*; qIs154(lag-2p:: myr::tdTomato)* V*; lag-2(cp193[lag-2:: mNeonGreenˆ3xFlag])* V.

### Strain maintenance and synchronization

Worm strains were maintained on nematode growth media (NGM) at 16°C unless otherwise specified. Populations were synchronized by bleaching according to a standard egg prep protocol (Stiernagle, 2006).

### Dauer formation by progressive starvation and crowding

To more precisely time the formation of facultative dauers, a large, mixed population of well-fed worms was transferred from an uncrowded growth plate to NGM plates seeded with ~70μL of an overnight culture of *E. coli* OP50 bacterial food. Plates were sealed with parafilm and placed at 25°C for the non-temperature-sensitive strains and 16°C for strains containing *daf-2(e1370), daf-7(e1372),* or *glp-1(bn18)* temperature-sensitive alleles. Plates were monitored daily for evidence of starvation (bacterial lawn depleted and worms dispersed). At 25°C this took ~2–4 days and at 16°C this took ~5–7 days The first day starvation was noted became day 0 of starvation. Early dauers were defined as coming from plates that were 5 days or fewer starved, while late dauers were defined as coming from plates that were at least 28 days starved.

### Brood size assays

All brood size assays were conducted at 16°C to match the necessary conditions of the temperature sensitive strains. After experimental treatment (see below), worms were singly picked to NGM plates seeded with ~70μL *E. coli* OP50. Egg-laying adults were subsequently passaged to a new plate once daily until egg laying stopped. Plates with progeny were kept at 16°C until the oldest worm on the plate had reached L4, after which they were counted. Schematic illustrating this method in Fig. 1E.

For never-dauer control brood assays, L4 animals of each strain were singled on Day 0. For brood after recovery from constitutive dauer formation, healthy plates of adults were egg prepped; eggs were rolled in M9 overnight at room temperature to obtain a population of arrested L1 larvae. Animals were dropped onto NGM plates seeded with OP50. Plates were sealed with parafilm and put at the restrictive temperature (25°C) to induce dauer formation. Early dauers that were constitutively formed came from plates 5 days or fewer after dauer formation; late dauers came from plates at least 28 days after dauer formation. For brood after recovery from progressive starvation, the above protocol was followed to induce dauer. Worms were then isolated by SDS treatment (see next section) and dauers were singly recovered to NGM plates seeded with OP50 at 16°C.

### Dauer isolation protocol

For plates with mixed populations, dauers were isolated with 1% SDS (Karp, 2018) After SDS treatment, the population of recovered animals was placed onto an unseeded NGM plate and the excess liquid was allowed to dry, after which animals were picked to a slide for imaging, were kept on the unseeded plate for the specified number of hours to evaluate short-term recovery, or picked to an NGM plate seeded with OP50 for recovery and brood measurement.

### Food removal experiments

A synchronized population of *daf-2(e1370); naSi2(mex-5p::H2B::mCherry::nos-2 3′UTR II)* L1s was added to NGM plates seeded with OP50 and were put at 25°C to induce constitutive dauer formation. After the specified number of hours (Fig. 2A), animals were rinsed off the seeded plate with M9, recovered to a 15mL conical tube, and were subsequently washed in M9 3–5 times and then rolled in M9 at room temperature for 20 minutes before being washed an additional time in M9 to clear bacteria. Washed animals were then transferred to peptone-free plates (recipe from (Eustice et al., 2022)) and returned to 25°C to continue to develop. Baseline imaging was done immediately after animals were washed off the food plates. Subsequent imaging done at the specified times (Fig. 2B). For Fig. 2B, animals were subjected to feeding/starvation for the indicated times and then kept at 25°C until the plate visibly had >50% dauers. Animals that appeared to be dauers under a dissecting scope were picked to image for the dauer endpoint of the experiment; only animals with dauer morphology were included in the final dataset. While removal of food in the L1 stage prior to the L1-L2d transition caused L1 developmental arrest, as expected, we found that removal of food after the L2d molt did not inhibit *daf-2(e1370)* animals from progressing through L2d or entering dauer.

### *glp-1(ts)* temperature shift experiments

Two temperature shift experiments used the temperature-sensitive allele of the Notch receptor *glp-1(bn18)*. To test for *glp-1* dependence during dauer (Fig. 4 A,B), two populations were used. As a control, an unstarved, uncrowded population was reared at 16°C, shifted to the restrictive temperature of 25°C for 24 hours, larval worms were singly picked to NGM plates seeded with *E. coli* OP50, and these plates were kept at 16°C until adulthood. As two test groups, worms that had been subjected to progressive starvation and crowding at 16°C (both early and late) were shifted to 25°C for 24 hours. After 24 hours, dauers were recovered by SDS isolation and singled to NGM plates seeded with *E. coli* OP50 and kept at 16°C until adulthood. Each plate was subsequently assayed for the presence of progeny.

To test for when *glp-1* dependence returns after dauer, *glp-1(bn18)* worms were put into dauer by progressive starvation at 16°C, shifted to 25°C overnight, and then SDS isolated, transferred to NGM plates without food and placed back at 25°C for an additional 4, 6, or 8 hours. At that time, the developmentally oldest/largest worms on the plate (the worms that exited dauer first, since dauer exit is asynchronous) were picked as single animals to NGM plates seeded with *E. coli* OP50 food and kept at 16°C until adulthood. After 4 days of recovery at 16°C, each plate was assayed for live larvae, and those that had offspring were scored as fertile. Plates that lacked larvae were imaged at 600x magnification in DIC and scored for the presence of embryos in the uterus (fertile) or an absence of gametes (sterile). Embryo retention appeared to be a phenotype of *glp-1(bn18*).

### Confocal imaging

All images were acquired at room temperature on a Leica DMI8 with an xLIGHT V3 confocal spinning disk head (89 North) with a 63× Plan-Apochromat (1.4 NA) objective and an ORCAFusion GenIII sCMOS camera (Hamamatsu Photonics) controlled by microManager. RFPs were excited with a 555 nm laser; GFP and mNG were excited with a 488 nm laser; mTurquoise was excited with a 445 nm laser; DAPI was excited with a 405 nm laser. Z-stacks through the gonad were acquired with a z-step size of 0.3 or 0.5 μm as noted. Worms were mounted on agar pads in M9 buffer with 0.01 M sodium azide (VWR (Avantor) Catalog Number 26628-22-8). Some samples were acquired with a 1.6X optical zoom, as indicated in the figure legends. Samples used for fluorescence quantification were acquired with the same laser power, exposure time, and z-step size within the datasets to be compared.

### Image analysis

Images were processed in FIJI89 (Version: 2.14.1/1.54f).

### Gonad length and body width measurements

From DIC confocal z-stacks, gonad length was measured from the distal most end of one gonad arm to the distal most end of the other gonad arm using the segmented line tool in FIJI following the general shape of the gonad. Body width was measured from cuticle edge to cuticle edge perpendicular to the gonad using the straight line tool in FIJI across the z-slice that captured the widest part of the body.

### Somatic gonad marker measurements

*Somatic gonad cell number* (Fig. 3J–K): The strain GS9692 expressing *arTi435(rps-27p::2xnls::gfp(flexon)::unc-54 3′ UTR)*; *arTi237(ckb-3p::Cre(opti)::tbb-2 3′ UTR)* (Shaffer and Greenwald, 2022) was specifically designed to activate enduring GFP expression in the cells of the somatic gonad. We examined this strain in early and late dauers after inducing dauer with progressive starvation and crowding (see above). We always see the expected two DTCs and 10 primordial gonad cells in dauers of any age. Variation in expression levels/tissue depth can sometimes complicate detection of the cell at the center of the gonad primordium (more orange cells in Fig. 3J). Landmarks were selected based on relative position with DTCs at the gonad tips and the next-most-distal fluorescent cells being the SS cells used for measuring.

*DTC measurement* (Fig. 3L–M): Strain NK2517 *qIs154(lag-2p:: myr::tdTomato); lag-2(cp193[lag-2:: mNeonGreenˆ3xFlag]* V*)* was imaged for the DTC membrane marker only at the stages specified in Fig. 3M. A sum projection through ten 0.3 μm z-steps capturing the superficial surface of the DTC was made, traced by hand in FIJI, and the area measured. We chose an L2 control for this experiment because, while the dauer larva is an alternative third larval stage, the worm’s reproductive system undergoes morphogenetic changes and changes in cell number during the normal L3 stage. These developmental events are arrested in dauer (Tenen and Greenwald, 2019), making L3 a confounding control for the dauer larvae reproductive system.

### Dauer recovery experiments

To measure gene expression during recovery, worms were isolated as described above (Dauer isolation protocol), and subsequently split such that some worms were imaged at 0 hours of recovery, and other worms were recovered to NGM plates without food (to isolate withdrawal of dauer maintenance conditions from food-specific cues) for the specified length of time.

### Reporters of *lag-2* measurement: Endogenously tagged LAG-2::mNG with coexpressed membrane TdTomato

Strain NK2517 *qIs154(lag-2p:: myr::tdTomato); lag-2(cp193[lag-2:: mNeonGreenˆ3xFlag]) V* was used to measure endogenously tagged LAG-2::mNG protein and a coexpressed transcriptional reporter of the *lag-2* promoter. All worms, including controls, were reared at 25°C. TdTomato has a half-time to maturation of one hour at 37°C (Shaner et al., 2004), while mNeonGreen has a half-time to maturation of fewer than ten minutes at 37°C (Shaner et al., 2013) (both will fold slower at worm-rearing temperatures). TdTomato signal is visible in adjacent neurons (Fig. 4 and Fig. 5) and the autofluorescent gut granules of the worm are visible in the channel of the endogenously tagged LAG-2::mNG. These structures were avoided as much as possible during projection, tracing, and measurement of signal.

Confocal z-stacks were acquired with a 0.3 μm step size. A sum intensity z-projection was created in FIJI consisting of 10 slices representing the superficial half of the cell (from the superficial cell surface to the cross-section of the nucleus). Either one or both DTCs from each worm were analyzed depending on if the gut obscured one. The DTC was hand-traced on the membrane marker RFP channel; that ROI was subsequently used to measure both the RFP (membrane) and GFP (endogenously tagged protein) channels, and slid off the DTC to the adjacent body of the worm to measure the background for both channels. Background subtracted measurements were thus obtained for both Mean Gray Value (mean pixel value in the ROI) and Integrated Density (total pixel value in the region = MGV*area). Integrated density for all samples was normalized to the mean integrated density at the 0h timepoint, so fold-change at 2h time point is shown.

### Reporters of *lag-2* measurement: Polycistronic histone reporter with coexpressed membrane mNeonGreen

We examined another pair of DTC-expressed markers: a faster-folding, membrane-localized *lag-2p::mNeonGreen::PLCδ1*^*PH*^ (*cpIs122,* (Linden et al., 2017)) and a histone H2B::mTurquoise2 (half-time to maturation of 33.5 minutes, (Goedhart et al., 2012)) that was knocked into the endogenous *lag-2* locus with a 2A peptide cleavage site (Medwig-Kinney et al., 2022). This second element will generate a polycistronic mRNA, the translation of which will produce one LAG-2 protein and one H2B::mTurquoise2, meaning that the histone reporter is under the same transcriptional and post-transcriptional, transcript-based regulation (e.g. by 3’ UTR-mediated repression by microRNAs or RNA binding proteins, transcript decay, etc.) as the endogenous *lag-2* gene. The membrane-localized mNeonGreen reporter, on the other hand, will simply come on when the *lag-2* promoter (including the TGF-β-responsive element described by (Pekar et al., 2017)) is active.

Confocal z-stacks were acquired with a 0.3 μm step size. A sum intensity z-projection through a 6–7 μm depth of the DTC (to capture the entire nucleus in z) was generated in FIJI, the DTC was hand-traced, and membrane fluorescence was measured for the GFP channel in that ROI. Both DTCs from each animal were analyzed in separate projections. A background measurement was made by sliding the DTC ROI onto the adjacent body of the worm. The nuclear histone signal was obtained using an ellipse tool tight around the nucleus in the same z-projection to measure on the CFP channel, and background for the nuclear signal was made by sliding that ROI onto the adjacent body of the worm. Background subtracted measurements were thus obtained for both Mean Gray Value (mean pixel value in the ROI) and Integrated Density (total pixel value in the region, MGV*area). Integrated density for all samples was normalized to the mean integrated density at the 0h timepoint, so fold-change at 2h time point is shown.

### Statistical analysis

All statistical analysis was done using GraphPad Prism version 10.4.2 for Windows, GraphPad Software, Boston, Massachusetts USA, www.graphpad.com. Specific tests specified in the relevant figure legends.

Statistical analysis of fold change shown in 5A, B, E, F; statistical analysis (unpaired t-test) of non-normalized integrated densities for these datasets show the same pattern of significance (that is, only LAG-2::mNG signal changes between 0h and 2h) as the fold change analysis.

## Data Availability

All relevant data and details of resources can be found within the article.
